# Evaluating the coating process of titanium dioxide nanoparticles and sodium tripolyphosphate on cucumbers under chilling condition to extend the shelf-life

**DOI:** 10.1038/s41598-021-99023-3

**Published:** 2021-10-13

**Authors:** Mahmoud Helal, Rokayya Sami, Ebtihal Khojah, Abeer Elhakem, Nada Benajiba, Amina A. M. Al-Mushhin, N. Fouda

**Affiliations:** 1 Department of Mechanical Engineering, Faculty of Engineering, Taif University, P.O. 11099, Taif, 21944 Saudi Arabia; 2 Department of Food Science and Nutrition, College of Sciences, Taif University, P.O. 11099, Taif, 21944 Saudi Arabia; 3 Department of Biology, College of Science and Humanities in Al-Kharj, Prince Sattam Bin Abdulaziz University, Al-Kharj, 11942 Saudi Arabia; 4 Department of Basic Health Sciences, Deanship of Preparatory Year, Princess Nourah Bint Abdulrahman University, P.O. Box 84428, Riyadh, 11671 Saudi Arabia; 5 Production & Mechanical Design Department, Faculty of Engineering, Mansoura University, Mansoura, Egypt

**Keywords:** Biochemistry, Biological techniques, Plant sciences, Nanoscience and technology

## Abstract

Cucumber is a highly perishable fruit, that can easily suffer from water loss, condensation, shriveling, yellowing and decay. The present investigation aim was to extending the shelf-life of cucumber using eco-friendly sodium tripolyphosphate and nano-material. Decay; hardness; succinate dehydrogenase activity (SDH); condensation and shriveling rates; and visual quality assessments of cucumbers fruits were evaluated during 21 days of storage period at 10 °C. There was a slight incidence of decay among (Chitosan/Titanium Dioxide Nanoparticles) CS-TiO_2_ and (Chitosan/Titanium Dioxide Nanoparticles/Sodium Tripolyphosphate) CS-TiO_2_-STP samples, which reported the lowest decay incidence 2.21% in CS-TiO_2_, while CS-TiO_2_-STP did not show any decay at end of storage period. CS-TiO_2_-STP recorded the lowest value in SDH activity 0.08 ∆OD min^−1^ mg protein^−1^. Very slight hardness, water condensation, and shriveling were detected in CS-TiO_2_ samples, while CS-TiO_2_-STP was the lowest compared with other SC samples and control. In general, CS-TiO_2_-STP treatment was found most potential to enhance the postharvest shelf life of cucumber throughout the storage period up to 21 day.

## Introduction

Cucumber (*Cucumis sativus* L.) is an essential crop that can be used in pickling and eaten fresh. It is excellent source of minerals, fibers, phytochemicals and vitamin^1^. The postharvest physiological disorder can reduce the overall quality, consumer acceptance, and storage shelf-life of cucumber; the recommended condition for storage of cucumber should be between 7 and 10 °C^2^. Postharvest problems include discoloration, surface pitting, sunken, dark colors, watery areas, chlorophyll breakdown, oxidation, spoilage, and fungal infections^3^. Several techniques and agrochemicals have been applied to reduce these problems to prolong the shelf-life by enhancing the bioactive compounds which delay the reactive oxygen species such as ozone treatment, hot water dips, organic salts, chilling, and chitosan (CS)^4–8^. The agrochemicals acceptability is decreasing due to the potential negative effects on health^9^. Thus, developing alternatives agrochemicals is essentials for research. Chitosan is biodegradable and can maintain color, prevent water loss, softening, delay ripening, fungal rot, and inhibit respiration^10,11^. Nano-coating processes can reduce physiological weight loss, decay, microbial growth, maintain firmness, protect the quality, inhibit the color changes, enzymes, and prevent senescence^12,13^. Nano-material films act as action items for enhancing flavors, nutrients against pathogen growth on the surface of foods^14,15^. Besides, they are non-toxic and well known for the food protection strategy in case of low quantities without any synthetic agents^16,17^. The American Food and Drug Administration US (FDA) reported that titanium dioxide nanoparticles (TiO_2_) are non-toxic and safe to be used in human foods and industry^18^. They have antibacterial activities, and effective film formability^19^. TiO_2_ has special morphology, physical–chemical, and mechanical characterizes to expand the shelf-life of numerous food items by dominating the gases influence and harmful rags^20,21^. The films can be printed with sodium tripolyphosphate (STP) to act as a crosslinker to stabilize the nanoparticle polymers in the nanoparticle formations^22^. Khojah el al.^22^ determined some of the postharvest physicochemical properties and microbial population effect of treated cucumbers such as chilling injury, CO_2_ respiration rate, chlorophyll contents, ascorbic acid content, total phenolic content, antioxidant activity, peroxidase enzyme activity and fungal populations. The combination of nano-coating materials such as titanium dioxide nanoparticles, chitosan, and sodium tripolyphosphate is rarely applied in cucumber preservation to prolong the storage period. Consequently, the current research was undertaken to evaluate the coating process effects such as decay rate, hardness, SHD activity, condensation, and shriveling rates on cucumbers under chilling condition for 21 days at 10 °C.

## Results

### Decay rate

**Figure 1:**
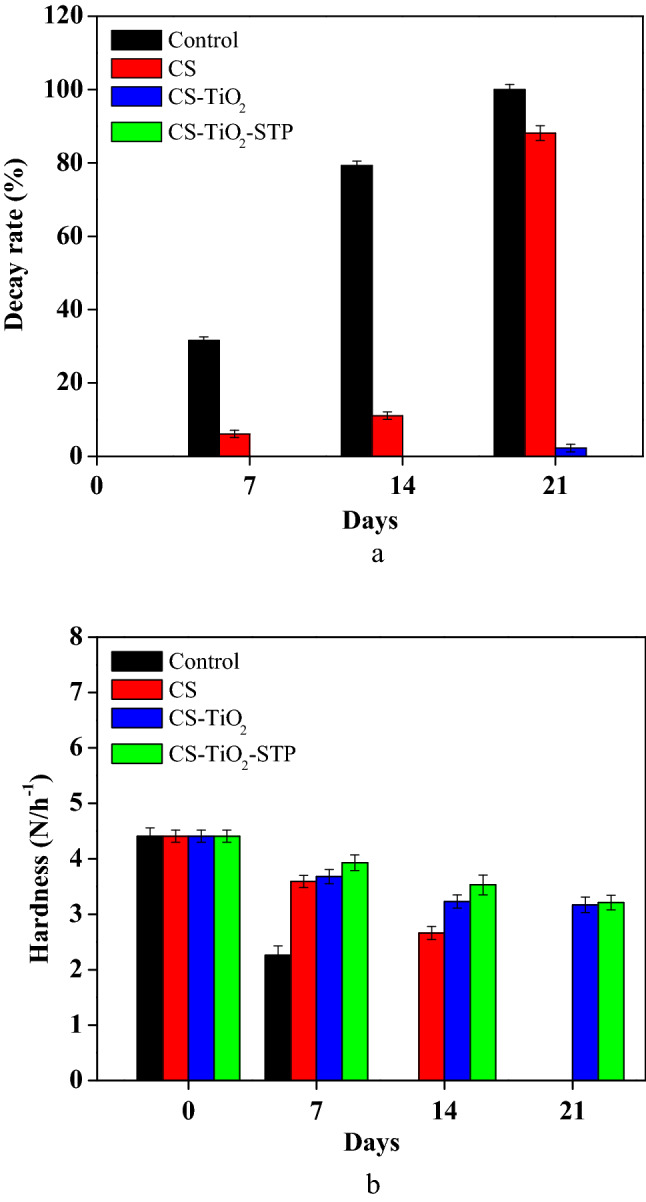
Effect of coating treatments of cucumber samples on decay rate (**a**), and hardness (**b**); (Control) refers to the treatment with distilled water, CS is the (Chitosan), CS-TiO_2_ is Chitosan/Titanium Dioxide Nanoparticles, while CS-TiO_2_-STP is (Chitosan/Titanium Dioxide Nanoparticles/Sodium Tripolyphosphate).

It was noticed that the decay started to appear in cucumbers after 7 days under the continuous condition at 10 °C. The control samples reported an increase of up to 31.52% decay, while the decay incidence of coated samples reached 6.11% in CS samples. At the same time, coated samples with nano-material or even with the addition of STP did not show any decay at all (Fig. 1a). Though, after one additional week of chilling storage, the control, CS samples reported 79.22% and 11.05% decay, respectively. After 3 weeks of chilling storage, the control samples were completely spoiled 100%, and CS samples reached 88.15% decay. There was a slight incidence of decay among CS-TiO_2_ and CS-TiO_2_-STP samples; which reported the lowest decay incidence 2.21% in CS-TiO_2_, while CS-TiO_2_-STP did not show any decay at all at the end of the storage period.

### Hardness loss

Figure 1b shows the effect of various coating treatments during the whole storage on the hardness loss of cucumber samples. The results indicated that the hardness showed a decreasing trend with increasing the storage time, where, it decreased from 4.41 to 2.26 N h^−1^ in the control samples. On the other hand, the hardness loss in CS cucumbers fruits decreased to reach 2.66 N h^−1^ on the 14th day. The coated cucumbers with CS-TiO_2_ (3.17 N h^−1^) and CS-TiO_2_-STP (3.21 N h^−1^) treatments were found to have greater hardness.

### SDH activity

Exposure of the coating process decreased from 0.22 to 0.08 ∆OD min^−1^ mg protein^−1^ for CS-TiO_2_-STP after the 21st day compared with the control samples (Table 1). There was a little difference in SDH activity in-between CS-TiO_2_-STP and CS-TiO_2_, which recorded 0.09 and 0.08 ∆OD min^−1^ mg protein^−1^ after the 21st days of the storage period, respectively. Though, SDH activity for CS was lower than in the control samples and recorded 0.11 ∆OD min^−1^ mg protein^−1^ after the 14th day of storage period and then spoiled upon continues storage.

**Table 1:** Effect of coating treatments of cucumber samples on SDH activity (∆OD min^−1^ mg protein^−1^).

Days	Control	CS	CS-TiO_2_	CS-TiO_2_-STP
0	0.22 ± 0.01^a^	0.22 ± 0.01^a^	0.22 ± 0.01^a^	0.22 ± 0.01^a^
7	0.16 ± 0.02^b^	0.17 ± 0.04^a^	0.14 ± 0.08^c^	0.13 ± 0.01^d^
14	–	0.11 ± 0.04^b^	0.12 ± 0.03^a^	0.10 ± 0.02^c^
21	–	–	0.09 ± 0.03^a^	0.08 ± 0.02^b^

*Values within a column (lowercase) are significantly different (p ≥ 0.05). The values in parentheses indicate (SD±) standard deviation.

(Control) refers to the treatment with distilled water, CS is the (Chitosan), CS-TiO_2_ is Chitosan/Titanium Dioxide Nanoparticles, while CS-TiO_2_-STP is (Chitosan/Titanium Dioxide Nanoparticles/Sodium Tripolyphosphate).

### Condensation and shriveling rates

**Figure 2:**
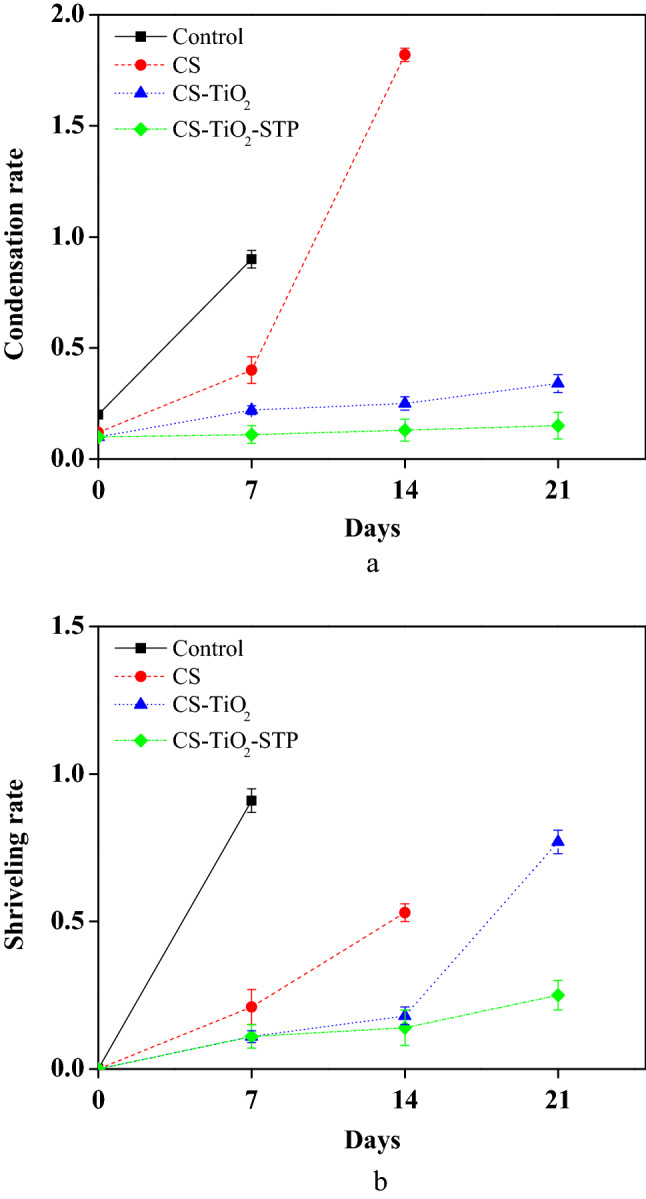
Effect of coating treatments of cucumber samples on condensation (**a**), and shriveling rates (**b**); (Control) refers to the treatment with distilled water, CS is the (Chitosan), CS-TiO_2_ is Chitosan/Titanium Dioxide Nanoparticles, while CS-TiO_2_-STP is (Chitosan/Titanium Dioxide Nanoparticles/Sodium Tripolyphosphate).

The obtained results showed that control and SC samples lost up to 11.34 and 1.82 losses of their initial weight after the 7th and the 14th days of storage at 10 °C, respectively. SC samples reached slightly to moderate levels on the 14th day of storage then spoiled upon continues storage (Fig. 2a). Condensation was minimal for CS-TiO_2_-STP and CS-TiO_2_, which recorded 0.15 and 0.34 losses after the 21st day of the storage period, respectively. Very slight water condensation was detected in CS-TiO_2_ samples, while no water condensation was detected in CS-TiO_2_-STP samples compared with the other SC samples and control as the condensed water resulted in a high decay rate. SC samples and control reported a more shriveling rate than the nano-coated cucumbers. During the chilled condition, shriveling rate among control samples was slight to moderate 0.91 on the 7th day and severe after upon continuous storage period. Besides, SC samples reported slight to moderate 0.53 on the 14th day and severe after upon continuous storage period, respectively. All nano-coated cucumbers reported lower rates with only a slight shriveling rate especially with CS-TiO_2_-STP samples which reached barely 0.25 shriveling rate (Fig. 2b). While CS-TiO_2_ samples reported a slight shriveling rate of 0.77 at the end of the storage period.

### Visual quality assessments

**Figure 3:**
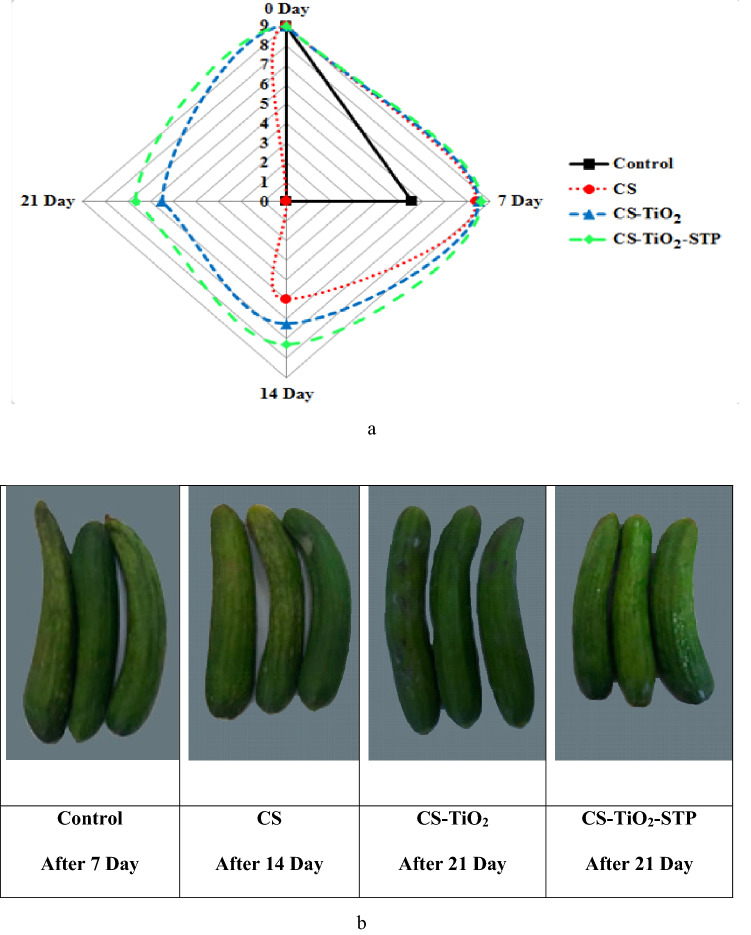
Effect of coating treatments of cucumber samples on visual quality assessments (**a**), and optical evaluation during the storage period (**b**); (Control) refers to the treatment with distilled water, CS is the (Chitosan), CS-TiO_2_ is Chitosan/Titanium Dioxide Nanoparticles, while CS-TiO_2_-STP is (Chitosan/Titanium Dioxide Nanoparticles/Sodium Tripolyphosphate).

The cucumber surface stored at 10 °C showed heavy damage, a watery surface pitting, chilling injury and became unacceptable to the panelists after the 7th day of storage especially for control samples, Fig. 3a. While, on the 14th day of storage, cucumbers dipped in CS solution reported a moderate surface pitting and light green externally with yellow spots (score of 9 to 4.99) then they were decayed upon contours storage. On the other hand, cucumbers coated with nano-materials presented better properties owing to the decay prevention which had slight or no watery surface pitting with good appearances for CS-TiO_2_ and CS-TiO_2_-STP, respectively. CS-TiO_2_ samples had better quality with a faint loss of green color of the peel (score of 9 to 5.49). In general, CS-TiO_2_-STP samples had the best visual ratings and appearance, indicating normal smell, freshness and firmness against the other treatments (Fig. 3b). CS-TiO_2_-STP treatment has the highest ones in this concern and protected the visual quality for 21 days of storage (score of 9 to 6.67).

In summary, the combination of chitosan (1%), titanium dioxide nanoparticles (1%), and sodium tripolyphosphate (2%) as coating solution (CS-TiO_2_-STP), great potential to preserve the physical–chemical properties and retained significantly higher sensory characteristics of cucumbers throughout the storage period. That combination evaluated parameters such as decay, hardness, SDH activity, and sensorial evaluations such as condensation, shriveling, and visual quality. The coating treatment had no side effects on the cucumber size, texture, and color and during the 21 days. Titanium dioxide nanoparticles with chitosan (CS-TiO_2_) preserved the cucumber too with lower quality than (CS-TiO_2_-STP). CS treatment maintained the cucumber for 14 days only, while the control was decayed after the 7th day of storage. It is recommended to preserve cucumber fruits by treating them with CS-TiO_2_-STP to extend the shelf-life to 21 days.

## Discussion

The Food and Drug Administration (FDA) recommended using nano-materials with low concentrations as 1 g kg^−1^ in drugs, cosmetics, and human foods which proved safety and as non-toxic^5,8,18^. Fruit decay is one of the serious problems in cucumber storage. All the coating treatments were greatly enhanced in reducing decay rate and so longer storage time compared to the control samples. Sami et al.^10^, found that chitosan treatment can inhibit the increase of oxidative enzymes such as peroxidase, polyphenol oxidase activities and decrease the physiological deterioration and the spoilage rate. Nano-coatings were found to be efficient methods to protect cucumber from decay. Several types of researches reported the efficient effects of chitosan coating and titanium dioxide nanocomposite material to delay the decay rate during storage on blueberry fruits and cantaloupes^7,8,21^. The coating that resulted in a decrease in decay rate can be related to washing off the pathogenic populations from the surface^6^. According to the previous results, the combination of TiO_2_ and STP promoted the decay signs and prolonged the shelf-life of cucumber till 21 days of storage period. The obtained results were in good agreement with Ebtihal et al.,^22^ and Shah et al.,^23^ who used sodium tripolyphosphate in food preservation due to the chemical, biochemical changes, anti-fungal activities; and enhancing the antioxidant, peroxidase enzyme activities, and the free radical scavenging capabilities. Fruit hardness is an essential parameter used to evaluate the quality. Eldib^8^, reported that post-harvest change in the hardness loss can be due to the enzymatic degradation of the components which is responsible for the structural rigidity. Nano-material coatings modified the internal gas compositions, reduced the oxygen, and elevated the carbon dioxide concentrations that explained the slower hardness loss of the samples^19,20^. Besides, the reduction in respiration rate of coated samples might be responsible for delaying the softening that resulted in the retention of hardness loss during the storage period^18,19^. It is well known that as the process of fruit ripening progresses and the depolymerization can occur with raises in pectinesterase activity^22^. The obtained results suggested that the hardness can be enhanced for more than 21 days if coated with CS-TiO_2_ and CS-TiO_2_-STP solutions. While other cucumbers had spoiled and the hardness measurement was incapable to be detected. SDH activity can be influenced by the storage conditions, commodity, and pH alteration as it can increase with the increase of pH^24,25^. SDH activity decreased in all cucumber samples during the whole storage period. Sami et al.,^10^ and Khojah et al.,^22^ were reported that the reduction of SDH activity can be due to high O_2_ and low CO_2_ gas concentrations due to the modification of the enzyme's structure. CS-TiO_2_-STP treatment was effectively influenced the SDH activity in cucumbers, which is linked with the previously published work of a lower respiratory rate after the addition of sodium tripolyphosphate which acted as a crosslinker to stabilize the nanoparticle polymers in the nanoparticle formations^22^. The rate of condensation is needed to be evaluated to enumerate the requirements for some mechanical dehumidifiers and the forced dehumidification measurements^26^. The main problem with cucumber preservations is water loss and condensation due to the high moisture content^22^. The extreme humidity atmosphere changes, air movement, dust on the sensor surfaces, and thermal radiation should be controlled to avoid disease developments, CO_2_ injury, and hypoxia fermentation which leads to tissue damage, warts, microbial spoilage, and influence food safety^6,27^. Mir and Beaudry^28^, reported that higher CO_2_ levels (5–10%) can cause yellowness and decay. Khojah et al.^22^, reported that the coating by nano-materials with the addition of sodium tripolyphosphate decreased the cucumber weight loss without obvious condensation. Przybytek et al.^29^, added soybean oil to the coating film which influenced the water-resistance characterizes. According to the obtained results, it is recommended to use nano-materials in the addition to sodium tripolyphosphate which can enhance the quality and reduce condensation rate. *Cucumis sativus* L. cucumber has a smooth peel. Although, during storage, it can be influenced by shriveling and developing of physiological peel blemishes, pitting, and warts due to moisture loss^1,22^. The current results were in agreement with Khojah et al.^22^, results who used sodium tripolyphosphate as a crosslinker to stabilize the nanoparticle polymers in the nanoparticle formations and to preserve cucumber quality, to enhance moisture loss, shriveling rate, peel disorders, yellowness, off-flavor, and to delay decay rate during chilling condition at 10 °C for 21 days. Visual quality assessments were the last parameters studied but they can be the most significant for the customers. The visual quality assessments such as texture, flavor, pulp and peel colors of treated and untreated cucumber fruits recorded a regular reduction with prolongation of storage period plus shelf life in the cucumber quality. Nano-coatings may reduce the deteriorative changes by decreasing desiccation, yellowing, and shriveling^5–8^. According to the previous reports, the visual quality was preserved by using edible coatings, chemical components such as sodium tripolyphosphate, and antibacterial agents such as nisin, thymol, and tween during the storage period^12–22^. Olawuyi and Lee^30^, reported similar results of the visual quality after treating fresh-cut cucumbers with chitosan and other packaging materials. While, Rageh and Abou-Elwafa^31^, reported an increase in the visual quality after treating cucumber with jasmine oil and dry yeast as a spray to prolong the shelf-life.

## Materials and methods

### Study period

All the experiments were performed during December 2020 to February 2021 at Taif University.

### Materials

Cucumber samples (*Cucumis sativus* L.) were obtained directly to the College of Sciences laboratory at commercial maturity. A total of 200 samples was used for conducting all coating processes. The sample was uniform in shape and size and without any external injury. These were washed with chlorinated water and allowed in a safe cabinet to air dry, while coating processes were carried out on the same day. TiO_2_ nanoparticles (30 nm), STP, CS (deacetylation degree of 85%), and acetic acid glacial (99% purity) as a preservative were from (Shanghai, China).

### Preparation of coating solutions

CS, CS-TiO_2_, and CS-TiO_2_-STP were prepared according to the author's previous report^21^, while distilled water was used as a coating solution for the untreated samples. A percentage of CS to TiO_2_ was 1%, while STP was 2% used in the current study.

### Coating processes and quality evaluations

Selected four sets of cucumbers were dipped in coating solutions for 10 min. Cucumbers were allowed in a safe cabinet to drain the excess liquids and chilled at 10 °C with 90–95% relative humidity (RH). The quality evaluation of cucumber samples were performed at interval of 7 days for 21 days of storage period.

### Decay rate

Each cucumber group was regularly indicated for the incidence of the microbial decay and the rate was calculated as a percentage of the total, according to the following Eq. (1)^7,8^:

1 $$ Decay\,Rate\left( \% \right) = \left( {1 - \frac{{V_{t} }}{{V_{o} }}} \right) \times 100, $$

(Where), ${V}_{t}$ is the infected cucumber at a certain degree of decay after time $t$ and ${V}_{o}$ is the raw cucumber. The visual determination was performed according to the following scores: = no decay (0%), slightly decay (25%), moderately decay (50%), and severely decay (> 50%)^9,21^.

### Hardness

The hardness was evaluated by a hardness meter (Model GY-1, China) with a range of 2–15 kg cm^−2^^32^.

### Succinate dehydrogenase activity

Succinate dehydrogenase activity (SDH) or respiratory Complex II is an enzyme complex, found in eukaryotes membrane or even inner mitochondrial cells. SDH enzyme activity was evaluated in cucumbers after changing the color from blue to colorless which depended on the sample enzymatic activity^33,34^. SDH enzyme activity was detected at an absorbance of 600 nm, remained linear for at least 3 min, and expressed as ∆OD per min^−1^ per mg protein^−1^.

### Condensation and shriveling rates

Cucumber quality evaluations during storage were recorded every 7 days for the physiological disorders on the surface such as condensation and shriveling which were ranged (from 0 to 3). (Where), 0 = no signs, 1 = slight, 2 = moderate, and 3 = severe according to Manjunatha and Anurag^35^.

### Visual quality assessments

Visual quality for each grouped cucumbers (coated and uncoated) was assessed for external and internal fruit appearance such as yellowing, pitting, pathogen infection, edible quality or any other disorder by (15) randomly selected untrained members in Taif University community. Changes that occurred during the storage period can influence marketing. Visual quality assessments were expressed by using 9-point hedonic scale [9 = represents excellent appearance, 7 = represents good, 5 = represents fair, limit of marketability, 3 = represents poor (useable), 1 = represents very poor (unusable)]. Visual quality assessments were applied every 7 days for 21 days^21,30^.

### Statistical analysis

The statistical analysis was carried out in-between the various treatments in triplicate during the whole storage period to analyze the differences. One-way analysis of variance (ANOVA) by (SAS 8.0 software, SAS Institute Inc., NC, USA). The significant difference of the data at P < 0.05 was established. Excel was used to calculate the mean ± standard deviations.
